# Altered Environmental Conditions Affect Responses to the Green Leaf Volatile *Z*-3-Hexenol in *Zea mays*

**DOI:** 10.3390/plants15030342

**Published:** 2026-01-23

**Authors:** Marie Engelberth, Jurgen Engelberth

**Affiliations:** Department of Biology, Health, and the Environment, The University of Texas at San Antonio, San Antonio, TX 78249, USA

**Keywords:** green leaf volatiles, abiotic stress, climate change, gene expression, plant protection

## Abstract

Green leaf volatiles (GLVs) are significant volatile signals that have been shown to protect plants against biotic and abiotic stresses, including insect herbivory and pathogen infections, as well as drought, cold, and heat stress. Since all these stresses are affected by climate change, GLVs provide an important target for research into their broad activities and their potential applications in agricultural settings. Therefore, to gain further insights into the protective properties of GLVs and their regulation under changing environmental conditions, we investigated whether climate-related changes alter the capacity to produce and the responsiveness to GLVs in *Zea mayss*, our model plant. Specifically, we studied the effects of limited nutrient supply, drought, and higher temperature. Neither significantly affected the capacity of plants to produce *Z*-3-hexenal as the first metabolite of the pathway, but elevated temperature increased *E*-2-hexenal production. We further identified changes in the effectiveness of plants to respond to GLVs under changing abiotic conditions by monitoring glucose levels and typical GLV-responsive genes covering metabolism, direct defense, indirect defense, and water stress. The results provide first evidence that plant responses to GLVs under defined environmentally challenging and stressful conditions are highly context-dependent and can vary substantially.

## 1. Introduction

Green leaf volatiles (GLVs) are rapidly produced by most plants in large quantities upon mechanical damage caused by biotic and abiotic stresses, including insect herbivory, necrotrophic pathogens, as well as cold, drought, and extreme temperatures [1,2]. When GLVs are perceived by undamaged parts of the same plant or by neighboring plants, they induce protective responses against these threats through signaling pathways that remain only partially understood. Aside from their immediate defensive effect, GLVs further appear to prime plants against future threats, enabling a stronger and/or faster response when these stresses ensue [1,3,4].

Over the last 20 years, research has provided extensive evidence to support these protective roles of GLVs in many plant species. In particular, their role in plant–insect interactions has been thoroughly investigated, and it has been demonstrated that GLVs provide significant protection against insect herbivory [1,3,4,5].

The biosynthesis of GLVs is essentially well established [1]. A lipoxygenase (LOX10 in maize [5]) inserts molecular oxygen at position 13 of linolenic acid, resulting in 13-hydroperoxy linolenic acid. A hydroperoxide lyase (HPL) then cleaves off a six-carbon unit as *Z*-3-hexenal (Z3al). Both enzymes are located within the chloroplast, and these reactions happen within seconds after mechanical leaf damage. The aldehyde is then reduced to *Z*-3-hexenol (Z3ol) and often esterified to an acetate by enzymes in the neighboring undamaged leaf cells, which requires a supply of NADPH and acetyl-CoA [1,6]. This transformation of the *Z*-3, but also their corresponding *E*-2 aldehydes, into the reduced forms is considered essential because of their potential toxicity to eukaryotic and prokaryotic cells [6,7]. However, while a variety of GLVs can be produced by plants, recent discoveries suggest that Z3ol appears to be the active compound. By creating mutant lines for specific hydrolases that remove the acetate from *Z*-3-hexenyl acetate, resulting in Z3ol, Cofer et al. [8] showed that this step is necessary to obtain biological activity.

GLVs were also found to protect plants against abiotic stresses, mainly those related to water stress, including drought, salt, heat, and cold stress [2]. While the specific signaling pathways triggered by GLVs for abiotic stress responses are not well understood, the results show that protection occurs on multiple levels and may include increased growth of stem and root systems, higher photosynthetic activities, increased antioxidative activities, the production of proteins that help maintain the structural integrity of the cells, reduced electrolyte leakage, and the storage of abscisic acid, which can rapidly become activated and induce protective responses [9,10,11,12,13,14,15]. Yet, it is still unclear why and how these compounds signal such widespread fortification against biotic and abiotic stresses. Nonetheless, due to their omnipotent protective properties, GLVs have been described as the plant’s multifunctional weapon [3].

However, most, if not all, of these experiments were performed either under constant and optimal laboratory conditions or, more recently, in field studies under more unpredictable conditions. Surprisingly, little is known about how plants respond to GLVs when they are perceived while being cultivated under stressful abiotic conditions like drought, reduced nutrient supply, and heat. To the best of our knowledge, no such investigation has been published to date, aside from one performed in our lab, which showed that even under cold stress conditions, GLVs are still active and can provide additional protection [15]. However, those plants were first cultivated under optimum conditions and only exposed to cold and GLVs for the relatively short duration of the experiment. Nonetheless, this initial experiment prompted us to further investigate plant responses to GLVs that are actually cultivated under altered, stressful abiotic conditions and to determine whether these conditions affect the perception of GLVs in maize as our model plant. We therefore hypothesized that plants cultivated under those stressful abiotic conditions will still be perceptive to GLVs but would also alter their responses. To test this hypothesis, we selected drought, heat, and limited nitrogen supply as abiotic stressors to study the responses of maize seedlings to GLVs by assessing potential changes in their capacity to produce GLVs and by analyzing transcript accumulation for selected genes. For this purpose, we chose four genes representing metabolism (*hexokinase* (*HK*), the starting point for glycolysis), water stress (*dehydrin* (*Deh*), which preserves structural integrity under water stress conditions), direct defense (*cysteine protease inhibitor* (*CPI*), which directly affects the attacking herbivore), and indirect defense (*terpene synthase 10* (*TPS10*), which produces compounds that indirectly affect the herbivore, for example, by attracting natural enemies). These genes have previously been shown to respond to GLV treatments by rapidly accumulating transcripts within 1 h [15,16,17,18,19]. Furthermore, we analyzed the glucose content of the plants as a marker for general metabolic activity [20,21]. The results clearly showed that changing environmental conditions have significant, but context-dependent, effects on the responsiveness of maize plants to GLVs.

## 2. Results

### 2.1. Abiotic Factors Have Limited Effects on the Capacity to Produce GLVs

**Figure 1:**
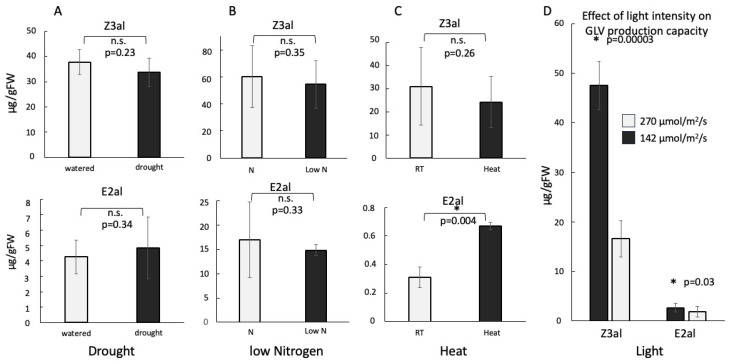
Effects of environmental stressors on aldehyde GLV production in maize (*Zea mays*). The *Y*-axis shows the amount of the measured aldehydes in microgram/gram fresh weight (µg/gFW). (**A**) drought stress. (**B**) low nitrogen stress. (**C**) heat stress. (**D**) light stress. Note that in this assay, no alcohols and esters were detected due to the total damage of the leaf tissue. * denotes significant differences (*t*-test, *p* ≤ 0.05). Non-significant differences (*p* ≥ 0.05) are denoted by n.s. *p*-values have been added for clarification. *N* ≥ 3; error bars represent standard deviation. Z3al, *Z*-3-hexenal; E2al, *E*-2-hexenal. N, treated with nitrogen-containing fertilizer; Low N, no nitrogen fertilizer added.

We first analyzed the capacity to produce GLVs, in particular *Z*-3-hexenal (Z3al) and *E*-2-hexenal (E2al), from leaf segments of maize plants that were cultivated under stressful abiotic conditions. For our analysis, we followed established procedures as described in [22]. We used drought stress, heat stress, nutrient deficiency, and higher light intensities as abiotic stressors. Surprisingly, we found no significant changes in the capacity to produce GLV aldehydes in drought- and nutrient-stressed maize plants (Figure 1A,B). A 5 °C increase in temperature also did not change Z3al production (31.0 ± 23.9 µg/gFW at 25 °C; 24.3 ± 16.8 µg/gFW Z3al at 30 °C) but had a significant effect on E2al production (Figure 1C). While control plants at 25 °C produced only 0.3 ± 0.07 µg/gFW, plants under heat stress produced more than twice as much E2al (0.7 ± 0.02 µg/gFW).

However, we found the most dramatic effect on the capacity to produce GLV aldehydes when we exposed plants to a higher light intensity (Figure 1D). By changing the photosynthetically active light from 140 µmol/m^2^/s to 270 µmol/m^2^/s for 24 h, we observed a 2.8-fold increase in Z3al production and a 1.42-fold increase in E2al. Since temperature and all other conditions were kept constant, the shift in GLV aldehyde production can therefore only be attributed to changes in light intensity.

### 2.2. Effects of Abiotic Stresses on Z-3-Hexenol-Induced Gene Expression and Glucose Levels

While abiotic stresses do not seem to affect the capacity of maize plants to produce GLVs, we further investigated how these stresses modulate the responses of plants to Z3ol by measuring transcript accumulation and glucose level. The selected genes represent a spectrum of what GLVs can activate and cover areas such as primary metabolism (*HK*), water stress (*Deh*), direct defense (*CystP*), and indirect defense (*TPS10*). For better comparison, we set the highest Z3ol-induced level at 100% for each biological replicate. We further selected one time point (60 min) for our analysis, since previous studies showed that the selected genes were all significantly upregulated at this time point [15,16,17,18,19]. All statistical analyses can be found in Table S1.

#### 2.2.1. Effects of Drought Stress on GLV-Induced Gene Expression and Glucose Levels

**Figure 2:**
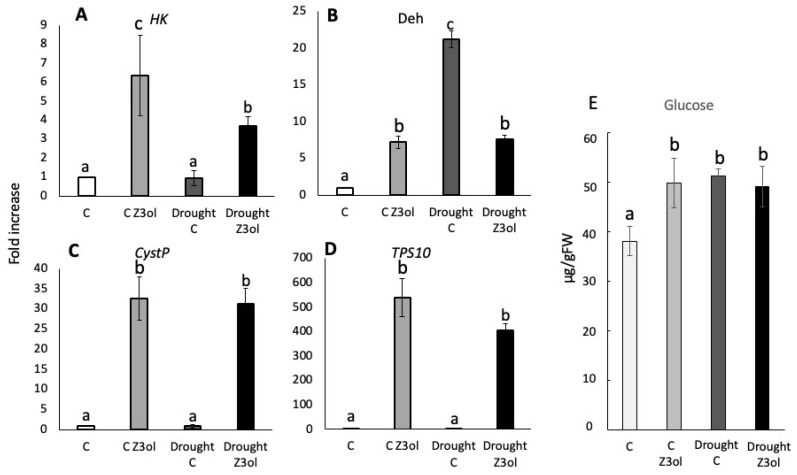
Transcript accumulation in leaves of maize (*Zea mays*) seedlings in response to drought treatment and *Z*-3-hexenol (Z3ol). Transcript accumulation was determined by the ^ΔΔ^Ct method and subsequent relative quantification (2^^^^ΔΔCt^). (**A**) Transcript accumulation of *hexokinase* (*HK*). (**B**) Transcript accumulation of *dehydrin* (*Deh*). (**C**) Transcript accumulation of *cysteine protease inhibitor* (*CystP*). (**D**) Transcript accumulation of *terpene synthase 10* (*TPS10*). (**E**) Glucose levels in microgram/gram fresh weight (µg/gFW)) in maize seedlings. C, unstressed control plants; C Z3ol, Z3ol-treated control plants; Drought C, drought-stressed control plants; Drought Z3ol, Z3ol-treated drought-stressed plants. Error bars represent standard error. (N ≥ 3). Different letters above each bar indicate statistical differences determined by two-way ANOVA analysis followed by Tukey tests where appropriate (*p* ≤ 0.05).

For *HK*, we found the highest levels of transcripts accumulation in control plants treated with Z3ol (6.6 ± 2.1-fold increase compared to corresponding control) (Figure 2A). However, *HK* transcript levels in Z3ol-treated drought-stressed plants accumulated to only 3.7 ± 0.5-fold. Control and just drought-stressed plants were not significantly different from each other with respect to *HK* transcript levels (Figure 2A).

Transcript accumulation for *Deh* showed a very different trend. While Z3ol-treated unstressed plants showed a 7.8 ± 0.9-fold increase compared to control, drought-stressed plants already showed much higher accumulation (21.4 ± 1.2-fold increase) (Figure 2B). Surprisingly, in Z3ol-treated drought-stressed plants, these levels were reduced within 1 h to the level observed in Z3ol-treated control plants (7.7 ± 0.6-fold increase).

*CystP* transcript accumulation was not affected by drought stress. Z3ol-treated control plants showed a 36 ± 5.3-fold increase, while Z3ol-treated drought-stressed plants reached a 31 ± 3.8-fold increase (Figure 2C). Transcript levels of *CystP* in control and drought-stressed plants were also not significantly different.

For *TPS10*, no significant differences were found between Z3ol-treated control (545 ± 77-fold increase) and Z3ol-treated drought-stressed plants (408 ± 26-fold increase) (Figure 2D). In addition, resting levels of *TPS10* in control and drought-stressed plants were also not significantly different (Figure 2D).

Glucose levels were also measured 1 h after Z3ol treatment. For unstressed control plants (C), we determined glucose levels of 38 ± 2.9 µg/gFW (Figure 2E). After Z3ol treatment (C Z3ol), glucose levels increased significantly to 50 ± 5.0 µg/gFW. In drought-stressed plants (C drought), we found glucose levels of 51 ± 1.2 µg/gFW, but did not measure a significant increase upon treatment with Z3ol (49 ± 4.1 µg/gFW).

#### 2.2.2. Effects of Low-Nitrogen Stress on GLV-Induced Gene Expression and Glucose Levels

**Figure 3:**
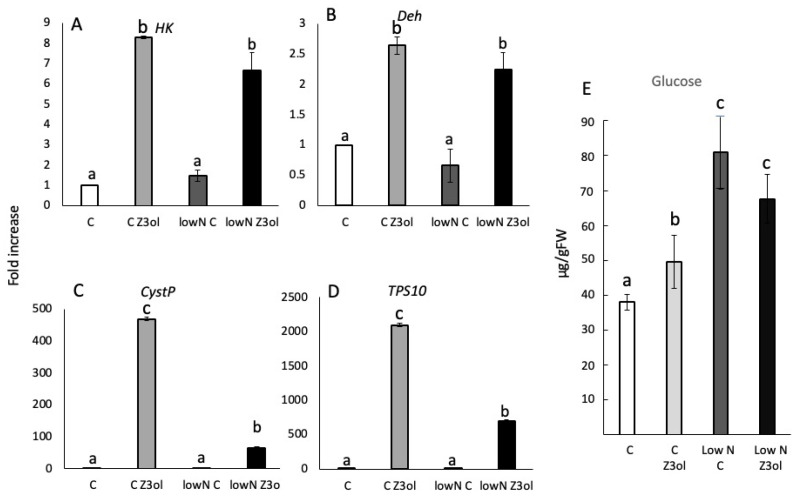
Transcript accumulation in leaves of maize (*Zea mays*) seedlings in response to low-nitrogen condition and *Z*-3-hexenol (Z3ol) treatment. Transcript accumulation was determined by the ^ΔΔ^Ct method and subsequent relative quantification (2^^ΔΔCt^). (**A**) Transcript accumulation of *hexokinase* (*HK*). (**B**) Transcript accumulation of *dehydrin* (*Deh*). (**C**) Transcript accumulation of *cysteine protease inhibitor* (*CystP*). (**D**) Transcript accumulation of *terpene synthase 10* (*TPS10*). (**E**) Glucose levels (microgram/gram fresh weight (µg/gFW)) in maize seedlings (second leaf) in response to low-nitrogen and Z3ol treatment. C, unstressed control plants; Low N C, low-nitrogen-stressed control plants; C Z3ol, *Z*3ol-treated unstressed control plants, Low N Z3ol, *Z*3ol-treated low-nitrogen-stressed plants. Error bars represent standard error. (N ≥ 3). Different letters above each bar indicate statistical differences determined by two-way ANOVA analysis followed by Tukey tests where appropriate (*p* ≤ 0.05).

*HK* transcript accumulation in control and low-nitrogen plants after Z3ol treatment was not found to be significantly different (8.2 ± 0.05-fold increase for control, 6.7 ± 0.1-fold for stressed plants) (Figure 3A). Likewise, resting transcript levels in control seedlings and low-nitrogen-stressed control seedlings were similar.

Similar results were also found for *Deh* transcript accumulation. Z3ol-treated control plants and Z3ol-treated low-nitrogen plants showed minor but not significant differences in transcript levels (2.6 ± 0.14- and 2.2 ± 0.28-fold increase, respectively) (Figure 3B). Likewise, no significant differences were found between control and low-nitrogen-stressed control plants (Figure 3B).

While *HK* and *Deh* showed no significant differences between control and low-nitrogen plants after Z3ol treatment, direct and indirect defense genes behaved very differently. *CystP* showed a 470 ± 5.4-fold increase in Z3ol-treated control plants but reached only a 66 ± 3.8-fold increase in Z3ol-treated low-nitrogen plants (Figure 3C). As before, control and low-nitrogen-stressed control plants showed only small but insignificant differences (Figure 3C).

Similar results were found for *TPS10* (Figure 3D). Z3ol-treated control plants were at a 2100 ± 31-fold increase, while low-nitrogen plants accumulated TPS10 to only 696 ± 17-fold after Z3ol treatment. Both control groups without Z3ol treatment were also not significantly different.

We also analyzed glucose concentration in low-nitrogen-stressed and control plants. In control plants without Z3ol treatment, we found glucose levels of 38 ± 2.2 µg/gFW, whereas with Z3ol treatment increased glucose levels to 46 ± 2.1 µg/gFW (Figure 3E). However, in low-nitrogen control plants, glucose levels were already significantly increased (70 ± 15.1 µg/gFW) compared to control plants. Z3ol treatment of low-nitrogen plants did not increase glucose levels, as described above for drought stress (68 ± 6.9 µg/gFW). These increases in glucose levels correspond to an 82% and 48% increase, respectively, when compared to the corresponding levels in fertilized control plants.

#### 2.2.3. Effects of Heat on GLV-Induced Gene Expression and Glucose Levels

To cultivate and treat maize seedlings under heat stress, we increased the growth room temperature from 25 °C (control) to 30 °C (heat), while keeping all other parameters constant. Under these conditions, we found contrasting patterns for *HK* and *Deh* transcript accumulation on the one side, and for *CystP* and *TPS10* on the other.

**Figure 4:**
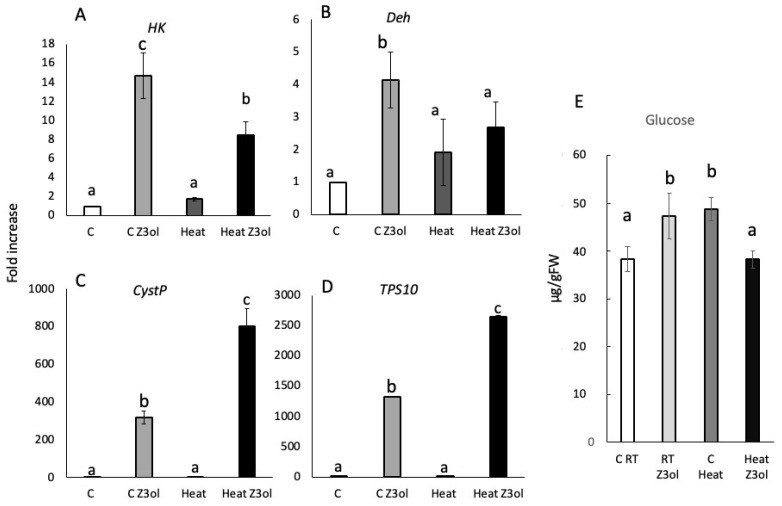
Transcript accumulation in leaves of maize (*Zea mays*) seedlings in response to heat treatment and *Z*-3-hexenol (Z3ol). Transcript accumulation was determined by the ^ΔΔ^Ct method and subsequent relative quantification (2^^ΔΔCt^). (**A**) Transcript accumulation of *hexokinase* (*HK*). (**B**) Transcript accumulation of *dehydrin* (*Deh*). (**C**) Transcript accumulation of *cysteine protease inhibitor* (*CystP*). (**D**) Transcript accumulation of *terpene synthase 10* (*TPS10*). (**E**) Glucose levels (microgram/gram fresh weight (µg/gFW)) in maize seedlings (second leaf) in response to heat and *Z*-3-hexenol treatment. C, unstressed control plants grown at room temperature (25 °C); Heat C, control plants grown under heat stress (30 °C); C Z3ol, *Z*3ol treatment of unstressed control plants; Heat Z3ol, *Z*3ol treatment of plants under heat stress. Error bars represent standard error. (N ≥ 3). Different letters above each bar indicate statistical differences determined by two-way ANOVA analysis followed by Tukey tests where appropriate (*p* ≤ 0.05).

For *HK* and *Deh*, Z3ol-treated control plants accumulated transcripts to 14.7 ± 2.4-fold for *HK* and 4.1 ± 0.9-fold for *Deh*, while Z3ol-treated heat-stressed plants only accumulated *HK* transcripts to 8.4 ± 1.4-fold and *Deh* transcripts non-significantly to 2.7 ± 0.8-fold compared to controls (Figure 4A,B).

On the other hand, direct and indirect defense genes (*CystP* and *TPS10*) showed a significant increase in transcript accumulation in Z3ol-treated heat-stressed plants compared to Z3ol-treated controls. We found an 802 ± 13-fold increase for *CystP* and a 2648 ± 13-fold increase for *TPS10* in Z3ol-treated heat-stressed maize seedlings, whereas Z3ol-treated control plants only accumulated a 318 ± 33-fold increase in transcripts for *CystP* and a 1330 ± 9-fold increase for *TPS10* (Figure 4C,D). Samples for control and heat-stressed plants showed no difference for either CystP or TPS10 (Figure 4C,D).

Likewise, glucose concentrations varied significantly between RT and heat treatments (Figure 4E). In RT controls, glucose levels were at 40 ± 3.3 µg/gFW, and Z3ol treatment increased this level to 47 ± 4.8 µg/gFW. While heat control plants already contained 48 ± 2.4 µg/gFW, and this level surprisingly decreased during Z3ol treatment to 38 ± 1.8 µg/gFW, thus making this the only treatment where glucose levels have been reduced after Z3ol treatment.

## 3. Discussion

The protective roles of GLVs in plant defense and abiotic stresses are well established [1,2]. GLVs provide rapid, direct protection by inducing defense-related and other protective genes and can also act as priming signals that enable faster and often stronger responses to subsequent stress. Priming by GLVs have been documented for biotic stresses, including insect herbivory and pathogen infection [1], as well as for abiotic stresses such as cold, salinity, and drought [2]. The broad spectrum of protective functions has led to their characterization as the plant’s multifunctional weapon [3].

This study investigated whether changing environmental conditions modulate plant responses to GLVs. Previous work has largely relied on plants grown and treated under standardized laboratory conditions or in field assays, in which environmental variation is uncontrolled and often unrecognized. Consequently, it remained unclear how plants perceive and respond to GLV while growing under these challenging environmental conditions. To advance understanding of this unexplored area, we therefore assessed responses to GLVs in maize seedlings that were cultivated under defined, environmentally stressful conditions and conducted all experiments within these altered settings. The results presented here demonstrate that plant responses to GLVs, measured as gene expression and glucose levels, are highly context-dependent and can vary substantially in different stressful environments.

We selected abiotic factors that are closely related to those encountered by plants during climate change, including drought, heat, and low nutrient supply. Interestingly, the capacity of maize seedlings to produce GLVs was not significantly affected by most of these altered growth conditions. Only increases in light intensity did upregulated the capacity to produce GLVs significantly. As we consider this as an unlikely natural occurrence—other than a sudden change in cloud cover—we decided not to pursue this aspect further unless we have more information on actual light intensity changes in natural environments and their potential physiology consequences for plants. Because GLVs have been shown to possess photoprotective properties, the observed increase may just reflect this activity [23]. However, we observed significant changes in the expression patterns of selected genes for all other tested stress conditions. We selected only one time point (1 h) for our analysis, because in maize all selected genes have been found to have significantly increased transcript accumulated when treated with GLVs at this time point [15,16,17,18,19]. Similarly, preliminary studies in our lab showed that GLV treatment caused the most significant changes in glucose levels 1 h after treatment. While we are aware of the limitation, this time point covers a phase of intense activities and provides relevant information about the changing responsiveness of maize plants to these treatments. However, we are also well aware that other factors like delayed or accelerated signaling may occur, as well as other factors like stress-specific regulatory networks that may also interfere with responses to Z3ol. Future studies should therefore look more contextually into these putative interactions and their potential consequences.

Drought is one of the most important factors that may harm plants in their natural environment, and its occurrence is often unpredictable [24,25]. Plants have therefore developed an array of protective measures, most of which are regulated by abscisic acid (ABA) [26]. However, in recent years, GLVs have also been found to activate responses that can help plants to better survive under drought conditions [2]. For GLV treatment under drought conditions, we found that responses were considerably affected. *HK* and *CystP* transcripts showed significant reductions, while *TPS10* expression was not affected. Most surprisingly, *Deh* transcripts, which are highly upregulated in drought-stressed control plants, were actually reduced by Z3ol to the exact same level observed in Z3ol-treated controls. This was unexpected, because in previous experiments with GLVs and cold stress, we found an additional increase in these gene transcripts, even when GLV treatment was performed under cold conditions [15]. Glucose levels, which are upregulated by GLVs within 1h, were already significantly higher in drought-stressed plants and did not respond to GLV treatment. This overall increase might be in part attributed to reduced water levels in drought-stressed plants, since glucose quantities are based on the fresh weight of plant material. Stomata may have been closed, reducing GLV uptake and, consequently, their activity [27,28]. However, *TPS10* did clearly respond to GLV treatment in drought-stressed plants, and the reduction in *Deh* transcript levels strongly suggests effective GLV uptake in order to cause the observed effects.

Low-nitrogen availability constitutes another important environmental factor. For example, increased rain fall can wash out nutrients from the soil, thereby depleting essential nutrients such as nitrogen and phosphate from plants [29,30,31]. Drought, on the other side, can limit nutrient uptake because water is required as a solvent for nutrient mobilization. Reduced nutrient supply has multiple physiological consequences for plants, with nitrogen deficiency being especially critical due to its central role in protein synthesis. In maize, Schmelz et al. [32] described the effects of low-nitrogen availability on volatile production and jasmonic acid accumulation after treatment with insect-derived elicitors. Jasmonic acid is the major plant hormone regulating plant defense responses to insect herbivory [33]. Surprisingly, nitrogen-starved plants produced significantly higher levels of jasmonic acid and volatiles after treatment with insect elicitors than well-supplied control plants. As glucose levels in low-nitrogen plants appear to be much higher than in fertilized control plants (Figure 3E), this additional supply may at least in part be responsible for the observed effects on volatiles by providing necessary metabolic resources. However, while these results provide interesting information about the defensive behavior of maize plants, they are not directly comparable to our findings, since those plants were treated with insect elicitors, while ours were treated with a bioactive volatile (Z3ol). It was also surprising to find a significant reduction in *TPS10*, a gene putatively involved in volatile production, as well as in *CystP*, which is a direct defense gene. While it is well established that GLVs do also induce rapid volatile production [34], no information is available on how this response might be affected by nutrient supply. In contrast to the monitored defense genes, *HK* and *Deh* were similar in their transcript levels between N and low N plants, suggesting a more targeted reduction in GLV-elicited defense responses.

Although average global temperatures are projected to increase only modestly, climate change is expected to increase the frequency of extreme temperatures. Such extreme events can impose substantial stress on plants, leading to a wide range of negative consequences. For our temperature stress study, we increased the ambient temperature in one of our growth chambers to 30 °C, while control plants were maintained at 25 °C. We recognize that temperatures exceeding 40 °C are common in some regions, particular in the southern United States. However, our objective was to demonstrate that even a modest change in ambient temperature can alter the plant’s response to GLVs. As shown above, even this relatively small increase already had significant consequences for the maize seedlings. Notably, the overall capacity to produce GLVs was not affected by this temperature increase; however, heat-stressed plants produced significantly higher levels of E2al compared to control plants. Fittingly, E2al, as well as other α, β-unsaturated carbonyls, have previously been described as a powerful inducer of abiotic stress-related genes, including heat stress-related transcription factors (*HFSA2* and *MBF1c*) [13]. Interestingly, both transcription factors are not induced by other GLVs, strongly implying a certain specificity of E2al in particular, but also other α, β-unsaturated carbonyls, with regard to heat protection.

In addition to altered GLV production, heat stress also had a significant effect on Z3ol-induced gene expression, which differed from maize seedlings grown under drought and low-nitrogen conditions. *HK* and *Deh* transcripts were significantly reduced at 30 °C, while *CystP* and *TPS10* transcripts were significantly upregulated. This response may also reflect changes in stomatal aperture, as under heat stress with sufficient availability of water, stomata are likely to open more widely to provide more cooling via increased water evaporation [35]. In addition, GLV uptake and transport appear to depend on open stomata [27,28]. However, this does not explain why *HK* and *Deh* transcripts are dramatically reduced. A reason for lower *HK* expression might lie in the fact that glucose levels in heat-stressed plants are higher, as was also found for drought stress and low-nitrogen plants. This suggests that glucose metabolism follows a certain trend by reducing *HK* transcript levels, which is considered to be the first committed step in glycolysis and is therefore a pivotal metabolic regulator by providing more substrates [18,19]. Therefore, additional HK protein production might not be necessary in order to increase metabolite production for energy or to make substrates available for other metabolic pathways, many of which are derived from glycolysis. And since glucose levels changed in the presence of Z3ol, in contrast to drought and low-nitrogen conditions, other, yet to be identified metabolic activities may also have become activated by GLVs.

While we have shown that changes in the abiotic environment significantly influence plant responses to GLVs, the ecological consequences of these modulated responses remain unclear. GLVs still appear to provide protection, but little is known about how insect herbivores or pathogens perform under these modified conditions. Furthermore, natural ecosystems rarely expose plants to single stressors. Instead, simultaneous or sequential stresses are common, and such interactions may profoundly influence the production, perception, and downstream effects of GLVs. Another important question concerns metabolism, particularly glucose-related metabolism, and how it might affect the general plant responses to both biotic and abiotic stresses. Although we detected significant changes in glucose levels, the implications of these changes remain unclear. Whether such metabolic adjustments enhance, constrain, or otherwise modulate volatile-mediated signaling and defenses still remains an open question. However, it is well established that glucose together with hexokinases, can serve as a sensor and regulator of various stresses in plants, as well as in other organisms [36]. Yet, the role of this system has not been explored in the context of plant defense responses, and much less so in plant responses to GLVs under challenging environmental conditions.

This study may therefore serve as a foundation for expanding research to include multiple stresses, diverse metabolic states, and diverse but well-defined ecological scenarios, thereby advancing our understanding of how GLVs function and how plants cope with an increasingly variable climate and changing environmental conditions.

## 4. Materials and Methods

### 4.1. Chemicals

*Z*-3-hexenol (Z3ol), *Z*-3-hexenyl acetate, and *E*-2-hexenal (E2al) were generously provided by Bedoukian Research Inc. (Danbury, CT, USA). *Z*-3-hexenal (Z3al) was purchased from Sigma-Adrich (St Louis, MO, USA). All solvents used were of analytical grade.

### 4.2. Plant Material

Maize (*Zea mays*, var. Kandy King, J.W. Jung Seed Co., Randolf, WI, USA) seeds were germinated in Sungro Horticulture Professional Growing Mix (Sun Gro Horticulture Canada Ltd., Seba Beach, AB, Canada) in pots with five seeds each. Plants were grown in a growth chamber under a 12 h photoperiod at 26 °C and 60% relative humidity for 4–5 days. Light intensity was set to ca. 142 μmol m^2^ s^−1^ for all experiments except light stress. After germination (development of the first leaf), plants were transferred to the respective abiotic treatment settings for a minimum of seven days and subsequently tested for GLV capacity and stress-related gene expression in response to Z3ol. We tested seedlings either at the late V_2_ or early V_3_ stage, with the fourth leaf emerging.

Treatment with Z3ol was performed in a plexiglass chamber (43 cm × 54 cm × 52 cm; Volume ≈ 120 L) by adding 1 μL of the pure compound to filter paper. Plants were treated for 1 h in all experiments.

### 4.3. Plant Treatments, Glucose Assay, Plant Volatile Collection, and Gene Transcript Accumulation

#### 4.3.1. Plant Treatment

Nutrient deprivation: Maize seedlings were grown in pots with vermiculite instead of soil. Control plants were treated with a nitrogen (N)-containing fertilizer (TPS nitrogen N-prime, Bellevue, WA 98009) according to the supplier’s recommendation, while stressed plants were grown without any addition of fertilizer. Upon first symptoms of N-depletion, such as red stem and reduced growth, as described in [24], we treated plants with Z3ol.

Heat: Pots with germinated maize seedlings (first leaf, day five) were transferred into plexiglass chambers (120 L volume). One chamber was heated to 30 °C with a heat pad placed underneath, while the other chamber was left at RT (25 °C; control). Plants were grown for seven days under these conditions and watered regularly. Temperatures were checked daily to ensure constant conditions. After seven days under these conditions, plants were treated with Z3ol within the chambers as described above.

Drought: Maize seedlings were grown as described above. After gemination and development of the first leaf (day five), control plants were watered regularly, whereas drought-stressed plants were not watered anymore. Plants were kept under these conditions for nine more days until first signs of drought stress were visible (e.g., curled leaves). At this stage, the water/soil ratio reached 0.5 ± 0.15 for drought-stressed plants and the water/soil ratio for well-watered maize seedlings was at 2.6 ± 0.2 (n = 6). Plants were then treated with Z3ol in a plexiglass chamber as described above.

Intense light: Ten-day-old maize seedlings were kept either under regular conditions as described in Section 4.2, with a light intensity of 142 μmol m^2^ s^−1^ (control), or were exposed to high light at 270 μmol m^2^ s^−1^. Leaf samples from high-light-treated plants were collected after 24 h, together with regular light-treated controls, and analyzed for GLV capacity.

#### 4.3.2. Glucose Assay

To analyze glucose, we used between 20 and 60 mg of maize leaf tissue, which was immediately shock-frozen in a 2 mL screw-cap vial in liquid N_2_. We then added 400 µL of 100 mM potassium phosphate (KPi) buffer, pH 6, and about 600 mg of beads (beads were added only once the buffer was frozen). The tissue was then homogenized in a Precellys homogenizer at 5600 for 25 s. After homogenization, we added 1 mL of dichloromethane and mixed the sample again in the Precellys homogenizer as described above. Samples were then briefly centrifuged at high speed (10,000× *g*) for 30 s for phase separation. The supernatant, which represents the aqueous phase, contained glucose among other polar compounds. We used 200 µL of this supernatant for our analysis. Fehling’s solutions A and B (Carolina Biological Supply (https://www.carolina.com(accessed on 10 December 2025); A # 862273, B # 862283) were mixed freshly by combining 1 mL of Solution A with 1 mL of Solution B, as suggested by the supplier. The Fehling solution was then diluted 1:10 with H_2_O and mixed thoroughly. We then added 500 µL of the Fehling solution to 200 µL of the glucose-containing aqueous phase and incubated the mixture for 10 min at 60 °C. A blank was prepared with KPi buffer and the Fehling solution. After the samples cooled down to RT, they were centrifuged at 10,000× *g* for 5 min and then analyzed using a spectrophotometer (Varian Cary 50 Bio) at 480 nm. A red precipitate may occur if glucose concentrations are high. If the glucose concentration was outside the linear range, the original extract should be diluted. We also analyzed other wavelengths (420 nm and 640 nm) but found that 480 nm provided the most reliable and reproducible results. Absolute quantification of glucose in the samples was determined by creating a standard curve with known amounts of glucose.

#### 4.3.3. Volatile Collection

For volatile collections, maize seedlings were taken from the different treatment groups described above on the same day that seedlings were also treated with Z3ol. GLVs (mainly Z3al and E2al) were collected from damaged leaf tissue (about 50 mg of freshly frozen (liquid N_2_) by homogenizing the plant material in a Precellys 24 (Bertin Technologies, Montigny-le-Bretonneux, France) with ceramic Zirmil^®^ beads (1.1 mm, about 600 mg; SEPR Ceramic Beads and Powders, Mountainside, NJ, USA) for 25 s at 6000 shakes per minute in a 2 mL screw-cap vial. Samples were then opened and immediately transferred to a 25 mL glass container sealed with a silicone-lined rubber septum in a screw-cap. Filters containing HayeSep Q80/100-absorption media (Supelco, Bellefonte, PA, USA) were inserted through a cut in the septum, and volatiles were trapped at a flow rate of 200 mL·min^−1^ for 1 h, as described previously [22]. Filters were then removed and eluted with 150 μL of dichloromethane. After adding the internal standard (nonyl acetate in dichloromethane at 1 µg/µL; 1 µg per sample), volatile samples were analyzed on a Varian model 3900 GC coupled to a Varian Saturn 2200 MS (ion trap) equipped with a split–splitless capillary injector system operating in electron impact mode (EI). The injection volume was 1 µL in split mode (1:10), and the injector temperature was set to 250 °C. The GC was programmed as follows: an initial temperature of 40 °C for 2 min, then temperature programmed at 15 °C/min to 250 °C. Separation of the volatile collections was performed on a fused silica capillary column (Equity™ 15 m × 0.25 mm inner diameter with a 0.25 µm film thickness of bonded methyl silicone (Sigma-Adrich, St Louis, MO, USA), with helium as the carrier gas at a flow rate of 1 mL/min. We set the solvent delay at 2.5 min, the transfer line to 260 °C, and the ion source at 180 °C. We used an ionization energy of 70 eV and a filament current of 10 µA. The scan rate was set to 2 scans/min, with a target total ion chromatogram (TIC) threshold of 20,000 counts. A scan range of 40–350 *m*/*z* was used. The equipment was tuned weekly, and Rf values were adjusted according to the manufacture’s specs. Data collection, storage, and subsequent analysis were performed on a computer using the Varian MS Workstation software (Version 6.6). The compounds were identified by comparison with authentic standards (retention time and fragmentation). Peak area determination was performed manually and compared to those of the internal standard. Final results are expressed as microgram/gram fresh weight (µg/gFW).

#### 4.3.4. Transcript Accumulation (qPCR)

To test for the effects of Z3ol on transcript accumulation of selected genes, we treated plants 1 h under the respective conditions outlined above. The second leaf was then cut off and snap-frozen in liquid nitrogen for RNA extraction. Similar segments from undamaged plants were used as controls. Leaves from three plants were pooled for one biological replicate, and three biological replicates were performed for each time point. We extracted the total RNA from ≤100 mg of ground leaf material using the PowerPlant^®^ RNA Isolation Kit containing DNAse (MO BIO Laboratories, Inc., Carlsbad, CA, USA), with the following modifications: frozen samples were homogenized in 2 mL screw-cap tubes containing 0.5 g of Zirmil microbeads and 200 µL of extraction buffer (PR1) for 20 s at 6000 shakes min^−1^ using a Precellys tissue homogenizer (MO BIO Laboratories, Inc., Carlsbad, CA, USA). After this initial homogenization step, we added 800 µL of PR1 and homogenized the samples for an additional 10 s at 6000 shakes min^−1^. The extract was then processed according to the manufacturer’s instructions.

We used the High-Capacity cDNA Reverse Transcript Kit (Applied Biosystems, Foster City, CA, USA) to synthesize the cDNA. We performed real-time PCR using the 7300 Real-Time PCR System (Applied Biosystems). PCR reactions were performed in a 20 µL volume containing 10 µL of SYBR Green PCR Master Mix (GLPBIO, Montclair, CA, USA), 0.2 µM each of forward and reverse primer, respectively, and cDNA equivalent to 25 ng of total RNA. Primer specificity was confirmed by melting curve analysis. Relative transcript levels were calculated using the 2^−ΔΔCT^ method [37], with *membrane protein PB1A10.07c* (*MEP*) as the reference gene [38]. We selected the following genes for analysis: *hexokinase 1* (*HK*) for metabolism [17,18,19,36], *terpene synthase 10* (*TPS10*) as a marker for indirect defenses [39,40], *cysteine protease inhibitor* (*CystP*) as a marker for direct defenses [18], and *dehydrin* (*Deh*) as a marker for drought stress [14,15].

### 4.4. Primer Sequences

*HK: F 5′-CGTACGTCGAAGAGGCCAGT, R 5′-GGAGGCAGGGCGAGTAGAAG*

*TPS10: F 5′-TGTGTCCACGGTCCAATGTT, R 5′-GTCCGCTGTCCTTGCAAAA*

*CystP: F 5′-GGACATGAGCTGGCGATTTT, R 5′-CAAGGAGCACAACAGGCAGA*

*MEP: F 5′-TGTACTCGGCAATGCTCTTG, R 5′-TTTGATGCTCCAGGCTTACC*

*Deh: F 5′-TGCTCGAGTACGAGATGTGG, R 5′-CTGATCATGTCCCAGACAGC*

### 4.5. Statistical Analyses

Expression data were analyzed by calculating the 2^−ΔΔCT^ method [37]. Statistical analyses were performed with ΔCT values due to their more normal distribution and linear scale [41]. At least three biological replicates were performed per experiment. Averages, standard errors (SE; for qPCR data), and standard deviations (SD; for glucose levels) were calculated for all samples (Microsoft Excel, Version 16.88). For pairwise comparisons, Student’s *t*-test was used (Microsoft Excel, Version 16.88), while for multiple comparisons, two-way ANOVA and Tukey’s test were applied (JMP statistical software, Version 17.2) (See Table S1).

## 5. Conclusions

The main goal of this study was to provide evidence that GLV perception in maize seedlings grown under altered stressful environmental conditions changes significantly. Drought, nutrient stress, and higher temperatures all modified the responses of maize seedlings to *Z*-3-hexenol, resulting in major changes in transcript accumulation of selected genes and glucose levels. At the same time, these altered environmental conditions did not change the capacity to produce GLVs. Changes in responses to Z3ol also seemed to be context-dependent, with very different outcomes observed for different stressors. These findings are likely to become more relevant as environmental conditions change all over the world due to climate change. GLVs, as the plant’s multifunctional weapon, may therefore become more relevant in the future as multi-protective agents that can help to maintain stable agriculture systems and regulate multitrophic interactions across diverse array of ecological niches. This study has been the first of its kind and may therefore serve as a starting point to further investigate how plants may be affected by GLVs and other protective signaling molecules in highly variable and constantly changing environments.

## Data Availability

Original data will be provided upon request by the corresponding author.

## Supplementary Materials

The following supporting information can be downloaded at: https://www.mdpi.com/article/10.3390/plants15030342/s1, Table S1: Results of statistical analysis.

## Abbreviations

The following abbreviations are used in this manuscript:

GLV	Green leaf volatiles
HK	Hexokinase 1
CystP	Cystein protease inhibitor
TPS10	Terpene synthase 10
MEP	Membrane protein PB1A10.07c
Deh	Dehydrin 2
Z3ol	*Z*-3-hexenol
Z3al	*Z*-3-hexenal
E2al	*E*-2-hexenal
