# Supplementary material for: Altered Environmental Conditions Affect Responses to the Green Leaf Volatile Z-3-Hexenol in Zea mays
Source: Plants (Basel). 2026 Jan 23;15(3):342. doi: 10.3390/plants15030342 (PMC12899973; doi:10.3390/plants15030342)
Supplement: Supplementary file 1 [file plants-15-00342-s001.zip › plants-4045295-supplementary.pdf]

Tukeys HSD test results for transcript accumulation (qPCR)

Hexokinase HK **Dehydrin Deh**

Drought Stress

treatments pair	Tukey HSD Q statistic	Tukey HSD p-value	Tukey HSD inferfence	treatments pair
Control vs drought Control	0.4189	0.8999947	insignificant	Control vs drought Control
Control vs C HOL	12.5521	0.0010053	** p<0.01	Control vs C HOL
Control vs drought HOL	7.0182	0.0048411	** p<0.01	Control vs drought HOL
Drought C vs C HOL	12.971	0.0010053	** p<0.01	Drought C vs C HOL
Drought C vs drought HOL	7.4371	0.0033914	** p<0.01	Drought C vs drought HOL
C HOL vs drought HOL	5.5339	0.0187239	* p<0.05	C HOL vs drought HOL

treatments pair	Tukey HSD Q statistic	Tukey HSD p-value	Tukey HSD inferfence	treatments pair
Control vs Heat Control	2.7318	0.2883946	insignificant	Control vs Heat Control
Control vs C HOL	46.1963	0.0010053	** p<0.01	Control vs C HOL
Control vs Heat HOL	25.5	0.0010053	** p<0.01	Control vs Heat HOL
Heat C vs C HOL	43.4645	0.0010053	** p<0.01	Heat C vs C HOL
Heat C vs Heat HOL	22.7681	0.0010053	** p<0.01	Heat C vs Heat HOL
C HOL vs Heat HOL	20.6963	0.0010053	** p<0.01	C HOL vs Heat HOL

Low Nitrogen

treatments pair	Tukey HSD Q statistic	Tukey HSD p-value	Tukey HSD inferfence	treatments pair
Control vs low N Control	0.6569	0.8999947	insignificant	Control vs low N Control
Control vs C HOL	9.5451	0.0010053	** p<0.01	Control vs C HOL
Control vs Low N HOL	7.3853	0.0035394	** p<0.01	Control vs Low N HOL
Low N C vs C HOL	8.8882	0.0010703	** p<0.01	Low N C vs C HOL
Low N C vs Low N HOL	6.7284	0.00624	** p<0.01	Low N C vs Low N HOL
C HOL vs Low N HOL	2.1598	0.467093	insignificant	C HOL vs Low N HOL

Cysteine Protease inhibitor CystP

Tukey HSD Q statistic	Tukey HSD p-value	Tukey HSD inferfence	treatments pair	Tukey HSD Q statistic	Tukey HSD p-value
17.0564	0.0010053	** p<0.01	Control vs drought Control	0.7009	0.8999947
5.5212	0.0189539	* p<0.05	Control vs C HOL	36.8403	0.0010053
5.5949	0.017669	* p<0.05	Control vs drought HOL	32.7443	0.0010053
11.5352	0.0010053	** p<0.01	Drought C vs C HOL	37.5412	0.0010053
11.4615	0.0010053	** p<0.01	Drought C vs drought HOL	33.4452	0.0010053
0.0737	0.8999947	insignificant	C HOL vs drought HOL	4.096	0.0770638

Tukey HSD Q statistic	Tukey HSD p-value	Tukey HSD inferfence	treatments pair	Tukey HSD Q statistic	Tukey HSD p-value
1.1804	0.8207433	insignificant	Control vs Heat Control	0.0101	0.8999947
8.9557	0.0010174	** p<0.01	Control vs C HOL	7.0427	0.0047426
4.2974	0.0630094	insignificant	Control vs Heat HOL	12.9372	0.0010053
7.7753	0.0025616	** p<0.01	Heat C vs C HOL	7.0326	0.0047815
3.117	0.2017448	insignificant	Heat C vs Heat HOL	12.9271	0.0010053
4.6583	0.0439788	* p<0.05	C HOL vs Heat HOL	5.8944	0.0133219

Tukey HSD Q statistic	Tukey HSD p-value	Tukey HSD inferfence	treatments pair	Tukey HSD Q statistic	Tukey HSD p-value
2.3835	0.3906573	insignificant	Control vs low N Control	0.037	0.8999947
11.574	0.0010053	** p<0.01	Control vs C HOL	28.539	0.0010053
8.7038	0.0012299	** p<0.01	Control vs Low N HOL	4.5637	0.0483157
13.9575	0.0010053	** p<0.01	Low N C vs C HOL	28.576	0.0010053
11.0873	0.0010053	** p<0.01	Low N C vs Low N HOL	4.6006	0.0465699
2.8702	0.254208	insignificant	C HOL vs Low N HOL	23.9753	0.0010053

Terpene Synthase 10 TPS 10

Tukey HSD inferfence	treatments pair	Tukey HSD Q statistic	Tukey HSD p-value	Tukey HSD inferfence
insignificant	Control vs drought Control	0.007	0.8999947	insignificant
** p<0.01	Control vs C HOL	7.5019	0.0032099	** p<0.01
** p<0.01	Control vs drought HOL	7.2204	0.0040713	** p<0.01
** p<0.01	Drought C vs C HOL	7.4948	0.0032302	** p<0.01
** p<0.01	Drought C vs drought HOL	7.2133	0.004096	** p<0.01
insignificant	C HOL vs drought HOL	0.2815	0.8999947	insignificant

Tukey HSD inferfence	treatments pair	Tukey HSD Q statistic	Tukey HSD p-value	Tukey HSD inferfence
insignificant	Control vs Heat Control	0.0013	0.8999947	insignificant
** p<0.01	Control vs C HOL	10.0385	0.0010053	** p<0.01
** p<0.01	Control vs Heat HOL	18.8911	0.0010053	** p<0.01
** p<0.01	Heat C vs C HOL	10.0372	0.0010053	** p<0.01
** p<0.01	Heat C vs Heat HOL	18.8898	0.0010053	** p<0.01
* p<0.05	C HOL vs Heat HOL	8.8526	0.0010995	** p<0.01

Tukey HSD inferfence	treatments pair	Tukey HSD Q statistic	Tukey HSD p-value	Tukey HSD inferfence
insignificant	Control vs low N Control	0.0183	0.8999947	insignificant
** p<0.01	Control vs C HOL	30.8575	0.0010053	** p<0.01
* p<0.05	Control vs Low N HOL	9.6331	0.0010053	** p<0.01
** p<0.01	Low N C vs C HOL	30.8758	0.0010053	** p<0.01
* p<0.05	Low N C vs Low N HOL	9.6514	0.0010053	** p<0.01
** p<0.01	C HOL vs Low N HOL	21.2244	0.0010053	** p<0.01

Heat

Hexokinase HK

Source	DF	Sum of Square (SS)	Mean Square (MS)	F Statistic (df_1,df_2)	P-value
Factor A - rows (A)	1	1.2744	1.2744	99.8923 (1,8)	8.522E-06
Factor B - columns (B)	1	0.113	0.113	8.8589 (1,8)	0.0177
Interaction AB	1	0.08345	0.08345	6.5409 (1,8)	0.03378
Error	8	0.1021	0.01276		
**Total**	**11**	**1.5729**	**0.143**		

Drought

Hexokinase HK

Source	DF	Sum of Square (SS)	Mean Square (MS)	F Statistic (df_1,df_2)	P-value
Factor A - rows (A)	1	1.2744	1.2744	99.8923 (1,8)	8.522E-06
Factor B - columns (B)	1	0.113	0.113	8.8589 (1,8)	0.0177
Interaction AB	1	0.08345	0.08345	6.5409 (1,8)	0.03378
Error	8	0.1021	0.01276		
**Total**	**11**	**1.5729**	**0.143**		

Low Nitrogen

Hexokinase HK

Source	DF	Sum of Square (SS)	Mean Square (MS)	F Statistic (df_1,df_2)	P-value
Factor A - rows (A)	1	1.6258	1.6258	66.2064 (1,8)	0.00003865
Factor B - columns (B)	1	0.01387	0.01387	0.5647 (1,8)	0.4739
Interaction AB	1	0.04871	0.04871	1.9835 (1,8)	0.1967
Error	8	0.1965	0.02456		
**Total**	**11**	**1.8848**	**0.1713**		

Dehydrin Deh **Cysteine Pr**

Source	DF	Sum of Square (SS)	Mean Square (MS)	F Statistic (df_1,df_2)	P-value	Source
Factor A - rows (A)	1	0.5592	0.5592	8.8219 (1,8)	0.01787	Factor A - rows (A)
Factor B - columns (B)	1	4.6501	4.6501	73.3597 (1,8)	0.00002663	Factor B - columns (B)
Interactio n AB	1	4.5705	4.5705	72.1027 (1,8)	0.00002836	Interactio n AB
Error	8	0.5071	0.06339			Error
**Total**	**11**	**10.2869**	**0.9352**			**Total**

Dehydrin Deh **Cysteine Pr**

Source	DF	Sum of Square (SS)	Mean Square (MS)	F Statistic (df_1,df_2)	P-value	Source
Factor A - rows (A)	1	0.5592	0.5592	8.8219 (1,8)	0.01787	Factor A - rows (A)
Factor B - columns (B)	1	4.6501	4.6501	73.3597 (1,8)	0.00002663	Factor B - columns (B)
Interactio n AB	1	4.5705	4.5705	72.1027 (1,8)	0.00002836	Interactio n AB
Error	8	0.5071	0.06339			Error
**Total**	**11**	**10.2869**	**0.9352**			**Total**

Dehydrin Deh **Cysteine Pr**

Source	DF	Sum of Square (SS)	Mean Square (MS)	F Statistic (df_1, df_2)	P-value	Source
Factor A - rows (A)	1	1.0947	1.0947	128.3832 (1,8)	3.316E-06	Factor A - rows (A)
Factor B - columns (B)	1	0.05884	0.05884	6.9003 (1,8)	0.03033	Factor B - columns (B)
Interaction AB	1	0.000505	0.000505	0.05922 (1,8)	0.8139	Interaction AB
Error	8	0.06822	0.008527			Error
**Total**	**11**	**1.2223**	**0.1111**			**Total**

otease Inhibitor CystP **Terpene Synthase 10 T[illegible]**

DF	Sum of Square (SS)	Mean Square (MS)	F Statistic (df_1,df_2)	P-value	Source	DF
1	2.4992	2.4992	1235.013 (1,8)	4.70E-10	Factor A - rows (A)	1
1	0.01164	0.01164	5.7526 (1,8)	0.04328	Factor B - columns (B)	1
1	0.005831	0.005831	2.8815 (1,8)	0.128	Interaction AB	1
8	0.01619	0.002024			Error	8
**11**	**2.5329**	**0.2303**			**Total**	**11**

otease Inhibitor CystP **Terpene Synthase 10 T[illegible]**

DF	Sum of Square (SS)	Mean Square (MS)	F Statistic (df_1,df_2)	P-value	Source	DF
1	2.4992	2.4992	1235.013 (1,8)	4.70E-10	Factor A - rows (A)	1
1	0.01164	0.01164	5.7526 (1,8)	0.04328	Factor B - columns (B)	1
1	0.005831	0.005831	2.8815 (1,8)	0.128	Interaction AB	1
8	0.01619	0.002024			Error	8
**11**	**2.5329**	**0.2303**			**Total**	**11**

otease Inhibitor CystP **Terpene Synthase 10 T[illegible]**

DF	Sum of Square (SS)	Mean Square (MS)	F Statistic (df_1,df_2)	P-value	Source	DF
1	1.0067	1.0067	274.5586 (1,8)	1.78E-07	Factor A - rows (A)	1
1	0.5286	0.5286	144.1475 (1,8)	2.136E-06	Factor B - columns (B)	1
1	0.5253	0.5253	143.261 (1,8)	2.186E-06	Interactio n AB	1
8	0.02933	0.003667			Error	8
**11**	**2.0899**	**0.19**			**Total**	**11**

'S 10

Sum of Square (SS)	Mean Square (MS)	F Statistic (df_1,df_2)	P-value
1.8956	1.8956	54.1342 (1,8)	0.00007936
0.000659	0.000659	0.01883 (1,8)	0.8942
0.000729	0.000729	0.02082 (1,8)	0.8888
0.2801	0.03502		
**2.1771**	**0.1979**		

'S 10

Sum of Square (SS)	Mean Square (MS)	F Statistic (df_1,df_2)	P-value
1.8956	1.8956	54.1342 (1,8)	0.00007936
0.000659	0.000659	0.01883 (1,8)	0.8942
0.000729	0.000729	0.02082 (1,8)	0.8888
0.2801	0.03502		
**2.1771**	**0.1979**		

'S 10

Sum of Square (SS)	Mean Square (MS)	F Statistic (df_1,df_2)	P-value
1.2894	1.2894	410.2428 (1,8)	3.69E-08
0.3546	0.3546	112.813 (1,8)	5.403E-06
0.3534	0.3534	112.4241 (1,8)	5.473E-06
0.02514	0.003143		
**2.0225**	**0.1839**		

Glucose Statistical Analysis

Var A/Var B	Control	**Low Nitrogen**
Control	x	x
Z-3-hexenol	x	x

Source	DF	Sum of Square (SS)	Mean Square (MS)	F Statistic (df_1,df_2)	P-value
Factor A - rows (A)	1	6555012.9	6555012.9	0.1733 (1,8)	0.6882
Factor B - columns (B)	1	3.407E+09	3.407E+09	90.0637 (1,8)	1.253E-05
Interaction AB	1	262548620	262548620	6.9395 (1,8)	0.02998
Error	8	302673456	37834182		
**Total**	**11**	**3.979E+09**	**361751393**		

Var A/Var B	RT	**Heat**
Control	x	x
Z-3-hexenol	x	x

Source	DF	Sum of Square (SS)	Mean Square (MS)	F Statistic (df_1,df_2)	P-value
Factor A - rows (A)	1	9147.2485	9147.2485	0.0008185 (1,9)	0.9778
Factor B - columns (B)	1	1318.711	1318.711	0.000118 (1,9)	0.9916
Interaction AB	1	307060739	307060739	27.4776 (1,9)	0.0005334
Error	9	100574669	11174963		
**Total**	**12**	**407645873**	**33970489**		

Var A/Var B	Control	**drought**
Control	x	x
Z-3-hexenol	x	x

Source	DF	Sum of Square (SS)	Mean Square (MS)	F Statistic (df_1,df_2)	P-value
Factor A - rows (A)	1	74141523	74141523	5.8357 (1,8)	0.04213
Factor B - columns (B)	1	122085713	122085713	9.6093 (1,8)	0.01467
Interaction AB	1	151177855	151177855	11.8992 (1,8)	0.008701
Error	8	101639326	12704916		
**Total**	**11**	**449044418**	**40822220**		

treatments	Tukey HSD	Tukey HSD	Tukey HSD
pair	T-statistic	p-value	inferfence
Control vs	1.5684	0.518419	insignificant
Control vs	8.5733	0.000219	** $p<0.01$
Control vs	6.4163	0.001608	** $p<0.01$
Control Z3	7.0049	0.000895	** $p<0.01$
Control Z3	4.8479	0.009134	** $p<0.01$
Low N Con	2.157	0.275071	insignificant

treatments	Tukey HSD	Tukey HSD	Tukey HSD
pair	Q statistic	p-value	inferfence
Control vs	5.0353	0.025917	* $p<0.05$
Control vs	5.4224	0.017293	* $p<0.05$
Control vs	0.0184	0.899995	insignificant
Control Z3	0.7614	0.899995	insignificant
Control Z3	5.055	0.025386	* $p<0.05$
Heat Contr	5.4408	0.016969	* $p<0.05$

treatments	Tukey HSD	Tukey HSD	Tukey HSD
pair	Q statistic	p-value	inferfence
Control vs	5.8652	0.013689	* $p<0.05$
Control vs	6.5494	0.007313	** $p<0.01$
Control vs	5.5156	0.019056	* $p<0.05$
Control Z3	0.6842	0.899995	insignificant
Control Z3	0.3496	0.899995	insignificant
Drought Co	1.0338	0.873772	insignificant
